# Childhood Disorder: Dysregulated Self-Conscious Emotions? Psychopathological Correlates of Implicit and Explicit Shame and Guilt in Clinical and Non-clinical Children and Adolescents

**DOI:** 10.3389/fpsyg.2022.822725

**Published:** 2022-03-09

**Authors:** Eline Hendriks, Peter Muris, Cor Meesters, Katrijn Houben

**Affiliations:** ^1^Department of Clinical Psychological Science, Maastricht University, Maastricht, Netherlands; ^2^Departement Sielkunde, Stellenbosch University, Stellenbosch, South Africa

**Keywords:** shame and guilt, implicit and explicit measurement, psychopathology, children, adolescents

## Abstract

This study examined psychopathological correlates of implicit and explicit shame and guilt in 30 clinical and 129 non-clinical youths aged 8–17 years. Shame and guilt were measured explicitly via two self-reports and a parent report, and implicitly by means of an Implicit Association Test (IAT), while a wide range of psychopathological symptoms were assessed with questionnaires completed by children, parents, and teachers. The results showed no differences of implicit and explicit shame and guilt between the clinical and non-clinical group, implying that dysregulation of these self-conscious emotions is not per definition associated with psychopathology. Correlational analyses indicated that self-reported explicit shame was positively associated with a broad range of internalizing psychopathology, while self-reported explicit guilt was associated with higher levels of anxiety and to some extent lower levels of externalizing psychopathology. Correlations with parent-rated shame and guilt revealed the same pattern of results but were in general weaker. Furthermore, implicit shame and guilt did not show significant correlations with the various measures of psychopathology. It can be concluded that the link between shame and guilt and psychopathology is complex, and partly dependent on the disorder under study and context-related factors defining the maladaptive nature of these self-conscious emotions.

## Introduction

The self-conscious emotions of shame and guilt drive people to behave in morally and socially appropriate ways. More specifically, these emotions are elicited when a person displays behavior or has a thought that violates some generally applicable social and moral rule or value (Tracy and Robins, 2004). Although shame and guilt arise to similar types of events, they are accompanied by different cognitive and behavioral manifestations. Shame is typically characterized by a negative evaluation of the self and hence prompts the person to show defensive and avoidance behavior, whereas guilt is more concerned with a negative evaluation of specific behavior and motivates the person to engage in reparative behavior by making apologies and engaging in attempts to correct the situation (Muris and Meesters, 2014). Thus, feelings of shame are more linked to discontentment with oneself and prompt a person to show submissive behavior, while feelings of guilt are more related to regrets about wrongdoing in a specific situation and motivate the individual to show more restorative behavior. Both emotions are normal phenomena that that help a person to maneuver effectively in social life. Meanwhile, there are clear individual differences in the degree to which a person reacts with shame or guilt, and sometimes these self-conscious emotions become dysregulated to such an extent that they start to interfere with the person’s daily functioning (Tangney et al., 1992b). For instance, when feelings of shame and guilt are experienced too intensely or frequently, interpersonal behaviors may become too accommodating (in the case of excessive guilt), avoidant, or submissive (in the case of excessive shame). Otherwise, if shame and guilt are not or insufficiently elicited after making a moral or social transgression, it is likely that the person does not show any corrective or reparative behavior, which can be interpreted as social insensitivity and can also cause interpersonal problems.

Indeed, research has shown that dysregulations of shame and guilt are associated with a variety of psychopathological outcomes (Tangney and Dearing, 2003). Most investigations on this topic have been conducted in adults and yielded a quite consistent pattern of findings: in particular shame has been demonstrated to be positively related to multiple forms of internalizing and externalizing problems (Tantam, 1998; Pallanti and Quercioli, 2000; Cavalera et al., 2016), whereas guilt is mainly associated with depression (Luck and Luck-Sikorski, 2021) or antisocial behavior for which this self-conscious emotion seems to be largely absent (Stuewig and Tangney, 2007). Relatively few studies have been conducted in children and adolescents, in spite of the fact that these self-conscious emotions already develop during the childhood years (Lewis, 2000). In general, the results of this research are in line with what has been found in adults, namely that young people who display higher levels of shame are also more prone to display higher levels of all kinds of psychopathological symptoms (Muris and Meesters, 2014). The link between guilt and psychopathology in young people is also less clear: there are indications that high levels of this self-conscious emotion are maladaptive, for example when guilt is experienced in a ruminative manner or is fused with feelings of shame, as appears to be the case in depressive disorders (Kim et al., 2011). There is evidence to suggest that working memory impairments associated with dysregulated self-conscious emotions play a prominent role in the formation of psychopathology (Cavalera et al., 2018). In contrast, low levels of guilt are thought to play a role in externalizing psychopathology of children and adolescents: lack of this self-conscious emotion would point at deficits in the development of empathy and conscience and as such be indicative for a lack of morality. As the current study is focused on children and adolescents, we will now discuss the relationship between the self-conscious emotions of shame and guilt and various types of child psychopathology in somewhat more detail.

### Depression

Most investigations on shame and guilt and internalizing problems in children and adolescents are concerned with depression. This research has generally demonstrated that both self-conscious emotions are positively correlated with depressive symptomatology, but that in particular shame plays a dominant role in this type of psychopathology. For example, De Rubeis and Hollenstein (2009) administered the Test Of Self-Conscious Affect for Adolescents (TOSCA-A; Tangney et al., 1991) as an index of shame proneness, and the Children’s Depression Inventory (CDI; Kovacs, 1985) as a self-report measure of depressive symptoms in a non-clinical sample of 141 children aged 11–16 years. The results showed a positive and statistically significant correlation of 0.57 between shame proneness and depression. At a 1-year follow-up, 46 children were re-tested with both questionnaires. It was found that shame proneness was moderately stable over time and that there was also evidence for a prospective relationship between shame levels at time 1 and depressive symptoms at 1-year follow-up (*r* = 0.29). De Rubeis and Hollenstein (2009) concluded that especially shame proneness is a significant predictor of depressive symptoms, both concurrently and prospectively. Other studies have confirmed the relevance of shame for the development and maintenance of depression in youths (e.g., Feiring et al., 2002; Tilghman-Osborne et al., 2008; Bennett et al., 2010).

### Anxiety Problems

Studies examining the relation between the self-conscious emotions of shame and guilt and anxiety disorder symptoms in children and adolescents are relatively sparse. One exception is a study by Muris et al. (2015) who examined relationships between shame and guilt proneness, anxiety-related vulnerability (i.e., the temperament characteristic of behavioral inhibition), and anxiety disorder symptoms in 126 non-clinical children aged 8–13 years, by administering a set of self-report questionnaires. The results indicated that both shame and guilt proneness were positively correlated with anxiety-related vulnerability and anxiety disorders symptoms. Additional analyses revealed that shame proneness remained a significant correlate of total anxiety and generalized anxiety symptoms, even after controlling for anxiety-related vulnerability and guilt. In contrast, guilt proneness did not remain significantly associated with anxiety pathology after controlling for the overlap with other variables. Thus, shame seems to be more relevant to symptoms of anxiety disorders in young people than guilt, a conclusion that has been confirmed in other more recent studies as well (Muris et al., 2018; Irwin et al., 2019; Broekhof et al., 2020; Sullivan et al., 2020).

### Borderline Personality Features

In adult studies, it has been demonstrated that individuals with borderline personality disorder (BPD) typically report higher levels of shame as compared to healthy controls or patients diagnosed with depression or anxiety (e.g., Rüsch et al., 2007). Although by definition a personality disorder diagnosis does not apply to people younger than 18 years, it has also been noted that stable borderline personality features (BPFs) can already be identified during childhood (e.g., Stepp et al., 2010). Interestingly, Hawes et al. (2013) examined whether youths with high levels of BPFs exhibit a similar shame-prone self-concept as adults with BPD. A community sample of youths aged 10–14 years was asked to fill in the Borderline Personality Features Scale for Children (BPFS-C; Crick et al., 2005) and complete an implicit association test (IAT) to assess shame proneness. Results indicated that the identity problems component of BPFs significantly predicted implicit levels of shame-proneness, although this was only the case among girls. This effect persisted after controlling for other psychopathological features such as hyperactivity/inattention, disruptive behavior problems, and anxiety/depression. Another investigation by Wall et al. (2021) examined the relation between explicit shame and BPFs in a sample of 184 adolescent inpatients (with an average age of 15 years) by administering three different self-report questionnaires measuring state and trait manifestations of shame and the BPFS-C. The results clearly indicated that adolescent inpatients with BPFs displayed statistically significantly higher levels of shame on all measures as compared to adolescent inpatients without borderline characteristics. Furthermore, it was found that BPFs explained unique variance in dispositional shame measures while controlling for gender, age, and concurrent internalizing and externalizing symptoms. Altogether, the available evidence indicates that implicit as well as explicit shame constitute an important feature of borderline features in young people.

### ADHD

Young people with ADHD have difficulties with inhibiting impulsivity which consequently leads up to breaking moral and social rules, but at the same time they are less thoughtful in monitoring their own actions (Barkley, 2015). Therefore, it can be assumed that they are less inclined to display self-conscious emotions. Evidence for this notion is provided in a study by Castagna et al. (2021) who asked caregivers of children (aged 5–12 years) to complete the Limited Prosocial Emotions Questionnaire (LPEQ; Castagna et al., 2020) and measures of externalizing symptoms. The limited prosocial emotions (LPEs) include lack of remorse/guilt, callousness/lack of empathy, unconcern about performance, and shallow/deficient affect. In their study, Castagna et al. (2020) looked at the association between various domains of LPEs and ADHD and other disruptive behavior problems such as oppositional-defiant disorder (ODD) and conduct disorder (CD). Their results showed that with the exception of callousness/lack of empathy, children with ADHD showed higher levels of all other domains of LPEs as compared to control children without a diagnosis. Most relevant within the context of the present paper, this appeared also true for lack of remorse/guilt, which is in line with the notion that self-conscious emotions are less prominent in young people with this type of externalizing problems (Tangney et al., 1996; Stuewig et al., 2010).

### Other Disruptive Behavior Problems

The results of the aforementioned study of Castagna et al. (2021) also showed that children with other disruptive behaviors tend to lack guilt and remorse. More precisely, a substantial percentage of the children with ODD/CD (39%) showed clear deficits in experiencing this type of self-conscious emotion (as compared to only 5% in children without a diagnosis). Similar results were obtained by Muris et al. (2015) who used data of the Achenbach System of Empirically Based Assessment (Achenbach and Rescorla, 2001) to examine the relationship between dysregulations in self-conscious emotions and psychopathology in a large sample of 1,000 clinically referred children and adolescents. It was found that lack of guilt was predominantly associated with the presence of oppositional defiant and conduct problems.

Another interesting study was conducted by Tangney et al. (1996) who administered age-appropriate variants of the TOSCA (Tangney et al., 1990) to measure shame and guilt proneness and scales of anger and aggression in a large sample of non-clinical participants (*N* = 1099) that also included children and adolescents (*n* = 729). Results again showed that guilt proneness was negatively linked to indices of disruptive behavior. More specifically, higher levels of this self-conscious emotion were associated with lower levels of aggression and more constructive ways of handling anger (e.g., corrective action, non-hostile discussion, and cognitive reappraisal). In contrast, shame proneness appeared to fuel maladaptive responses to anger, as became evident by positive correlations with aggression and malevolent intentions. Other studies have been conducted on the link between self-conscious emotions and disruptive and even delinquent behavior, and in general the results have shown that guilt is negatively and shame is positively related to such problems (Bennett et al., 2005; Hosser et al., 2008; Thomaes et al., 2008; Bear et al., 2009; Heaven et al., 2009; Kochanska et al., 2009; Stuewig et al., 2015).

### Methodological Considerations

In sum, an increasing number of studies have examined the links between shame and guilt proneness and psychopathology in children and adolescents. The overall conclusion of this accumulating evidence is that excessive and intense levels of shame are positively linked to both internalizing (i.e., anxiety, depression, and BPFs) and externalizing (i.e., anger, aggression, and delinquency) problems. In contrast, guilt seems to be more adaptive: this emotion is assumed to guide prosocial behaviors and has been shown to be negatively related to externalizing (i.e., ADHD, ODD, and CD) problems (Muris and Meesters, 2014). As such, studies that have been conducted with children and adolescents have revealed the same pattern of findings as documented in adult populations (Tangney et al., 1992a). One methodological shortcoming of previous empirical work on the link between self-conscious emotions and psychopathology in youth is that many researchers have relied on non-clinical samples (for exceptions, see Van Tijen et al., 2004; Hawes et al., 2013; Wall et al., 2021). The inclusion of clinically referred children and adolescents would make it possible to make a comparison between a clinical and non-clinical sample and to learn more about the relationship between self-conscious emotions and psychopathology. Given current consensus that most mental health problems represent a continuum (e.g., Krueger et al., 2018), one would expect that (a) aberrant levels of self-conscious emotions would be more prominent in clinical samples, and (b) similar relations between self-conscious emotions and symptoms of various disorders will be found within clinical and non-clinical samples.

Another methodological consideration pertains to the assessment of shame and guilt in previous research with children and adolescents. Most of the studies have relied on variants of the TOSCA (Tangney et al., 1989), which is a scenario-based instrument measuring self-conscious emotions on an explicit level. This means that young participants picture themselves in a situation in which some moral or social standard is violated and then have to make a conscious judgment about the intensity of shame and guilt experienced in that situation. However, it may also be that shame or guilt are implicitly triggered. Implicit tests do not rely on a person’s introspection, but use techniques that monitor non-conscious and often automated influences on one’s judgment, behavior, and motivation. The Implicit Association Test (IAT; Greenwald et al., 1998) is a commonly used instrument to measure implicit emotions and attitudes and has shown to have superior psychometric properties as compared to other implicit paradigms (Bar-Anan and Nosek, 2014). The IAT uses reaction time measurements to determine the relative strength of implicit associations between concepts (i.e., self vs. others) and attributes (e.g., ashamed vs. proud, guilty vs. innocent), based on the notion that quicker processing speeds indicate the presence of stronger associations. So far, the IAT as an implicit measure of shame and guilt has been predominantly used in adult populations with PTSD (Bockers et al., 2016), personality pathology (Rüsch et al., 2007; Ritter et al., 2014; Spitzer et al., 2021), and criminal offending (Nentjes et al., 2017). With children and adolescents, only one study can be found: the earlier described investigation by Hawes et al. (2013) explored the relationship between implicit shame and borderline personality features (BPFs) in young people aged 10–14 years. The IAT used in this study assessed the relative strength of the implicit association between the target concept of “self” (versus “other”) and the attribute concept of “shame” (versus “pride”). Results indicated that implicit shame was significantly associated with identity problems, being a core component of BPFs, in particular among girls.

### The Present Study

The present study was conducted to further examine the relations between the self-conscious emotions of shame and guilt and various types of psychopathological symptoms in non-clinical and clinically referred children and adolescents. We adopted a multi-informant, multi-method approach in which youths aged 8–17 years not only completed standardized scales for measuring shame and guilt proneness at a conscious, explicit level, but also conducted an implicit test to assess the susceptibility to experience self-conscious emotions at a more automatic level. Specifically, children and adolescents completed the Test Of Self-Conscious Affect (TOSCA-C; Tangney et al., 1990) and the Brief Shame and Guilt Questionnaire for Children (BSGQ-C; Novin and Rieffe, 2015) as explicit measures of habitual shame and guilt proneness, whereas the Implicit Association Test (IAT; Greenwald et al., 1998) was used to measure the relative strength of implicit association between the concept of “self” and both self-conscious emotions. The parents of all youths as well as the teachers of the non-clinical youths also completed a standardized questionnaire for rating children’s shame and guilt levels from their perspective. Furthermore, various questionnaires were administered in both groups for measuring various types of psychopathology, including internalizing (i.e., anxiety, depression, and BPFs) and externalizing (i.e., ADHD, aggression, ODD and CD) problems. It was hypothesized that clinically referred children and adolescents would display more dysregulated levels of shame and guilt, both on explicit and implicit measures, as compared to non-clinical youth. Further, it was expected that both explicit and implicit shame would be positively related to both internalizing and externalizing problems, whereas explicit and implicit guilt was anticipated to be negatively related to externalizing symptoms.

## Materials and Methods

### Participants

#### Non-clinical Population

Youths of the non-clinical population were recruited from four primary schools in the south-eastern part of the Netherlands and one secondary school in the adjacent Flemish part of Belgium. All children in the three oldest age groups of the primary schools and adolescents in the third and fourth year of social sciences classes in the secondary school were invited to participate. Parents and children (if aged 12 years onwards) received a letter, which provided them with background information about the study, along with a consent form. Eventually, (the parents of) 129 children (47 boys and 82 girls) responded positively to this invitation, which means that the participation rate was 59%. Children had a mean age of 12.61 years (*SD* = 2.39, range 9–17 years). Most children were from original Dutch (*n* = 76) or Belgium (*n* = 50) descent. One child was of American origin, one child was of Filipino descent, and one child had a Dutch and Belgium nationality.

#### Clinical Population

Participants of the clinical group were children and adolescents who were referred to Youz Maastricht between 2017 and 2020. Youz Maastricht is an outpatient diagnostics and treatment facility for youth with mental health problems. During the standard intake procedure parents received a letter with background information about the study and an informed consent form. The attrition rate was quite high in the clinical sample (13 children, 30%), with the sensitive topic and considerable amount of effort to complete all measures being the most frequently mentioned reasons. Eventually, 30 children (14 boys and 16 girls) completed the full procedure. These children had a mean age of 11.03 years (*SD* = 2.13, range 8–15 years) and were all of Dutch descent. All children were subjected to an extensive diagnostic procedure by licensed psychologists and psychiatrists. Data from multiple sources (i.e., interviews with the child, parents, and the teacher; psychological assessment; psychiatric examination; observations at the facility, at home, and/or at school) were used to establish a DSM-5-based clinical diagnosis following the Longitudinal, Expert, All Data (LEAD) procedure (Spitzer, 1983). The majority of children had attention-deficit/hyperactivity disorder as primary diagnosis (i.e., 21 children, 70% of the total group): 16 children were diagnosed with the combined type and 5 children with the predominantly inattentive type. Three children were diagnosed with autism spectrum disorder (ASD; 10.0%), three children had an anxiety disorder (10.0%), two children had a mood disorder (6.7%), and one child had an eating disorder (3.3%). About one quarter of the children were diagnosed with multiple disorders (i.e., 26.6%, e.g., ADHD and ASD), and more than half of them (i.e., 57%) faced additional difficulties like relational problems (i.e., parent-child relational problems or disruption of family by separation or divorce) and academic or educational problems.

### Procedure

Measures of the non-clinical group were administered by a Ph.D. student and a senior researcher at school in small groups, whereas children and adolescents of the clinical group were individually tested by the Ph.D. student at the clinical facility. All participants were informed that participation was on a voluntary basis and that they were free to withdraw from the study at any time. It was emphasized that there were no right or wrong answers, but that it was the answers most true for the respondent that we were interested in. Children were able to ask the researchers for help if they had any trouble understanding the questions. Administration of the questionnaires took between 30 and 45 min, depending largely on the age of the participants. After completing the set of questionnaires, children of the non-clinical group completed the Implicit Association Test (IAT) in small groups of up to 5 students in a separate classroom at school. Youths of the clinical group were tested individually and thus completed the IAT alone in the presence of the researcher. Parents of both groups and teachers of the non-clinical group completed a more limited set of questionnaires at home or at school. They both completed a brief measure of shame and guilt proneness (the BSGQ) and a broad index of psychopathology (ASEBA), while only parents completed additional measures of ADHD and anxiety/depression symptoms. Following completion of the whole procedure young children in the primary schools received a small present, while adolescents in secondary school were given a voucher for the cafeteria of their school. Youths of the clinical group received a cinema ticket.

### Assessment

#### Shame and Guilt Implicit Association Test

In the current study the IAT (Greenwald et al., 1998) was used to index the relative strength of implicit associations between the target concept of “self” (vs. “other”) and the attribute concepts of “shame” and “guilt”. The premise of the task is that word stimuli will be classified faster when target and attribute concepts match better for the participant and thus reflect a stronger automatic association. For example, this means that an individual whose self-concept is highly associated with shame will record faster reaction times to presentations in which the target concept “self” and the attribute concept “shame” are assigned to the same response key, compared with the pairing of “self” and the contrasting attribute concept “pride.” The IAT was programmed in Inquisit (version 3.0.6.0) by Millisecond Software and administered on a 14-inch laptop. During the task, target words referring to “self” (me, myself, first name of the participant, last name of the participant) and “other” (they, someone, them, and friend) were presented in the center of the screen, while attributes representing the contrast between the categories of “shame” (i.e., bad, stupid, loser, and worthless) and “pride” (i.e., fantastic, smart, topper, and amazing), and the categories of “guilt” (i.e., fault, wrong, naughty, and disobedient) and “pride” (i.e., good, fine, sweet, and obedient) were displayed in the upper corners of the screen throughout all testing blocks (see Figure 1 of the Supplementary Material for an example). Thus, participants completed the IAT two times, once for measuring the implicit association with shame (versus pride) and once for assessing the implicit association with guilt (versus pride). This was done in a counterbalanced order, with half of the participants completing the shame (versus pride) IAT before the guilt (versus pride) IAT, and the other half completing the tasks in reversed order.

**FIGURE 1:**
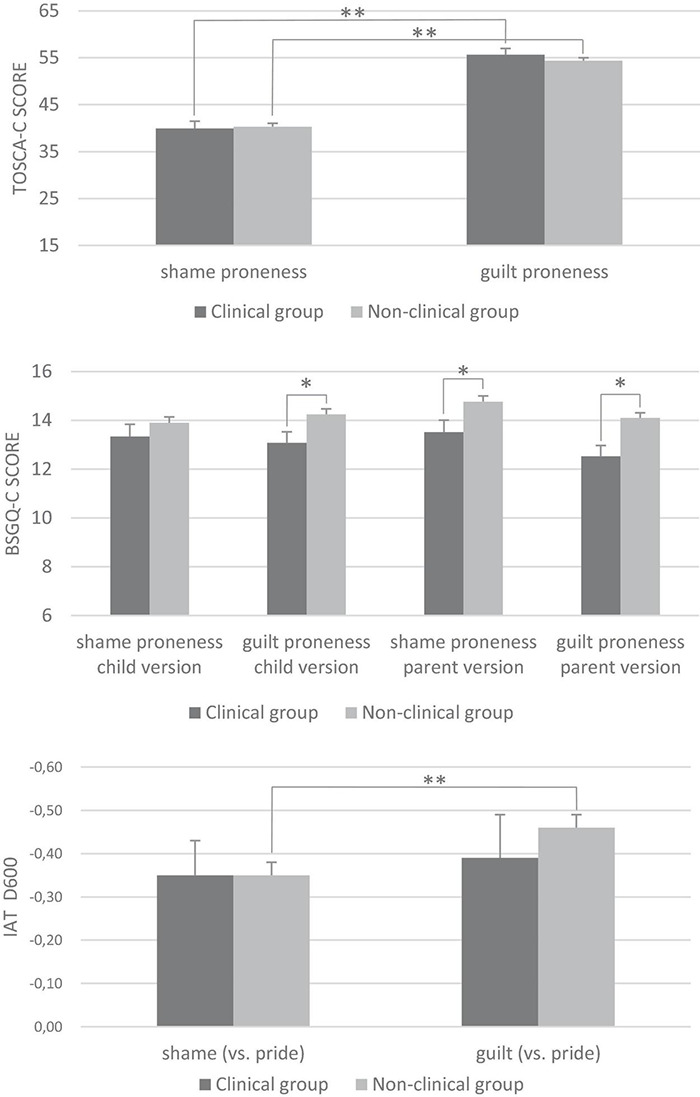
Mean scores (with standard error bars) on various explicit and implicit measures of shame and guilt in the clinical and non-clinical group. BSGQ-C, Brief Shame and Guilt Questionnaire for Children; IAT, Implicit Association Test; TOSCA-C, Test of Self-Conscious Affect for Children. The IAT D600 represents the contrast between the self- conscious emotions and pride. The negative values indicate that in general there was a stronger association with the attribute ‘pride’ than with the self-conscious emotions of ‘shame’ and ‘guilt’. *p < 0.05, ^**^p < 0.001.

The structure of the IAT is shown in Table 1. Both IATs consisted of five blocks. For the shame IAT, the procedure was as follows. In block 1, participants were required to classify words into the attribute categories “shame” versus “pride,” and in block 2 into the target categories “self” versus “other.” This was done by pressing response keys E (for words belonging to the category shown on the left side of the screen) and I (for words belonging to the category shown on the right side). In block 3, participants classified words belonging to one target category and one attribute category using one response key (e.g., “self” or “shame” on the left) and words belonging to the other target category and attribute category using the other response key (e.g., “other” or “pride” on the right). In block 4, the response assignment of the target categories was reversed (e.g., when block 2 included the categories “self” on the left and “other” on the right, block 4 included “self” on the right and “other” on the left). In block 5, participants then performed the reversed combination of target and attribute categories (e.g., “other” or “shame” on the left; “self” or “pride” on the right). The response assignment of the target and attribute categories was counterbalanced across participants so that half the participants classified “self” together with “shame” in the first combination block while the other half of the participants started out by classifying “self” together with “pride.” In blocks 3 and 5, target trials that contained either a “self” or “other” word were randomized with attribute trials that presented either a “shame” or “pride” word. Feedback was only given in the practice trials after making an error by showing a small red cross in the middle of the screen. The guilt IAT followed the same procedure as the shame IAT, but now with the attribute categories “guilt” versus “pride.” Completion of both IAT tasks typically required 10 min in total.

**TABLE 1:** Structure of the Implicit Association Test (IAT).

Block	No. of trials	Function	Items assigned to left-key response	Items assigned to right-key response
1	16	Attribute practice	Shame/guilt	Pride
2	16	Target practice	Self	Other
3	16	First pairing practice	Self + shame/guilt	Other + pride
3	48	First pairing test	Self + shame/guilt	Other + pride
4	16	Reversed target practice	Other	Self
5	16	Second pairing practice	Other + shame/guilt	Self + pride
5	48	Second pairing test	Other + shame/guilt	Self + pride

*IAT, Implicit Association Test.*

Implicit Association Test scores for both shame and guilt were calculated with the D600 scoring algorithm (Greenwald et al., 2003). Following the formula presented by Greenwald et al. (2003), IAT effects were calculated using both practice and test trials, by subtracting the average reaction time for the combination of “self” and “shame”/“guilt” (versus “other” + “pride”) from the average reaction time for the combination of “self” with “pride” (versus “other” + “shame”/”guilt”). Trials with latencies greater than 10,000 ms were excluded from the calculation of the D600 score, and participant data were also discarded if more than 10% of latencies were faster than 300 ms. Further, in line with the scoring algorithm, error penalties (600 ms) were given in case of an incorrect response and results were standardized at the level of the participant. A higher (positive) D600 IAT score indicated a stronger implicit association between the self and the self-conscious emotions of shame or guilt.

#### Questionnaires of Self-Conscious Emotion

The first questionnaire used to measure self-conscious emotions was the Dutch version of the *Test Of Self-Conscious Affect* (TOSCA-C; Tangney et al., 1990), which contains 15 brief scenarios that young people are likely to encounter in daily life. Each scenario is followed by a number of possible responses for which applicability can be rated on a 5-point scale (with 1 = ‘very untrue’ and 5 = ‘very true’). Thus, for all scenarios, children are asked to imagine themselves in the given situation and rate their response to indicate the likelihood of reacting in the manner indicated. For the purpose of this study, only TOSCA-C items measuring shame and guilt proneness were used; the subscales externalization, alpha pride, beta pride, and detachment/unconcern were not employed. The internal consistency coefficients of the TOSCA-C shame and guilt subscales have been shown to be good. More specifically, in previous research, the shame proneness subscale displayed Cronbach’s alphas of 0.77 for adolescents and 0.78 for children, while alphas for the guilt proneness subscale were 0.81 for adolescents and 0.83 for children (Tangney et al., 1996).

The *Brief Shame and Guilt Questionnaire for Children* (BSGQ-C; Novin and Rieffe, 2015) is the second questionnaire used to measure shame and guilt on an explicit level. Like the TOSCA-C, the BSGQ-C consists of brief vignettes of which six scenarios assess shame (e.g., “You are walking in the middle of a busy shopping street and stumble. All your books and pens fall out of your bag and roll down the street”), and six scenarios measure guilt (e.g., “You quickly eat the last biscuit and now there is no biscuit left for your friend”). For each scenario, children are asked to rate how much shame or guilt they would feel using a 3-point scale (1 = ‘not at all,’ 2 = ‘a little,’ and 3 = ‘a lot’). In the present study, we also used the parent and teacher version of the BSGQ-C which are similar to the version used for children, but here items ask observers to rate experienced levels of shame and guilt of children and adolescents from their perspective. The internal consistency of the shame and guilt subscales has been demonstrated to be good, with Cronbach’s alphas of 0.80 and 0.76, respectively (Novin and Rieffe, 2015).

#### Psychopathology Scales

The *Achenbach System of Empirically Based Assessment* (ASEBA; Achenbach and Rescorla, 2001) comprises 112 items addressing emotional and behavioral problems in youths. Parents and teachers completed the parent and teacher version of the scale, i.e., the Child Behavior Checklist (CBCL) and Teacher Report Form (TRF), while children themselves filled out the self-report version, i.e., the Youth Self-Report (YSR). All informants have to indicate on 3-point scales the extent to which each item applies to the child or adolescent (with 0 = ‘not,’ 1 = ‘sometimes,’ and 2 = ‘often’). The Achenbach scales address two main types of psychopathological symptoms that occur in young people. The first type is internalizing which reflects emotional symptoms (e.g., anxiety, depression, and somatic symptoms) and the other type is externalizing which refers to behavioral problems (e.g., aggression and truancy), and these so-called ‘broad band’ scales were also used in the present study. Previous research has shown that the CBCL, TRF, and YSR are reliable and valid scales for measuring psychopathological symptoms in young people and that this is also true for the Dutch versions of these questionnaires (Verhulst et al., 1996, 1997a,1997b).

The Dutch shortened version of the *Revised Child Anxiety and Depression Scale-25* (RCADS-25; Chorpita et al., 2000; Muris et al., 2002) is a 25-item scale that measures symptoms of anxiety (e.g., “I worry when I think I have done poorly at something”) and depression (e.g., “I feel sad or empty”) in children and adolescents. In the present study, children and parents were asked to indicate the frequency of symptoms on a 4-point Likert scale (with 0 = ‘never,’ 1 = ‘sometimes,’ 2 = ‘often,’ and 3 = ‘always’). The RCADS-25 has acceptable reliability in clinic and school-based samples (Muris et al., 2002; Ebesutani et al., 2012; Klaufus et al., 2020), and this appears also to be true for the parent version of the scale (Ebesutani et al., 2011).

The Dutch *ADHD Questionnaire* (AQ; Scholte and Van der Ploeg, 2005) is a scale consisting of 18 items that cover the three behavioral aspects of ADHD, namely attention-deficit (e.g., “is easily distracted”), hyperactivity (e.g., “talks continuously”), and impulsivity (e.g., “has difficulty to wait his or her turn”). Children are asked to rate the degree to which they experience these problems using a 5-point Likert scale (with 1 = ‘not,’ 2 = ‘occasionally’ or ‘incidentally,’ 3 = ‘regularly’ or ‘monthly,’ 4 = ‘often’ or ‘weekly,’ 5 = ‘very often’ or ‘daily’). The parent version of the AQ is similar to the child version but items are phrased from the parents’ perspective. In the present study, only the total score of ADHD was used. The AQ has good reliability and validity.

The *Reactive-Proactive Aggression Questionnaire* (RPQ; Raine et al., 2006) is a 23-item self-report instrument that measures reactive (e.g., “I get angry when threatened”) and proactive (e.g., “I had fights with others to show who was on top”) aggression. The items are rated on a 3-point Likert scale with 0 = ‘never,’ 1 = ‘sometimes,’ or 2 = ‘often.’ In this study, the total score of reactive-proactive aggression was used. Previous research has shown that the RPQ, including the Dutch version, is a reliable and valid questionnaire for assessing both forms of aggression in youths (Raine et al., 2006; Cima et al., 2013).

The short version of the *Borderline Personality Features Scale for Children* (BPFS-C; Sharp et al., 2014) comprises 11 items addressing BPFs in young people, such as affective instability (e.g., “My feelings are very strong. For instance, when I get mad, I get really really mad. When I get happy, I get really really happy”), identity problems (e.g., “How I feel about myself changes a lot”), and negative relationships (e.g., “I feel very lonely”). Children are asked to rate the items using a 5-point Likert scale ranging from 1 = ‘not true at all’ to 5 = ‘always true.’ The short BPFS-C has shown adequate psychometric properties in a sample of adolescent inpatients (Sharp et al., 2014) and non-clinical adolescents from the community (Fossati et al., 2019).

### Statistical Analyses

The Statistical Package for Social Sciences (SPSS, Version 26) was used. Descriptive statistics and reliability coefficients (Cronbach’s alphas) statistics for various questionnaires were computed. Independent samples *t*-tests were conducted to evaluate gender differences and to compare guilt and shame scores between the clinical and non-clinical groups on both implicit and explicit measures. Paired samples *t*-tests were used to compare guilt and shame scores within each group. Finally, correlation analyses were conducted in order to examine associations among study variables and in particular between explicit and implicit indices of shame and guilt on the one hand and various types of psychopathological symptoms on the other hand.

## Results

### General Findings

Before addressing the main research questions of the present study, a number of general findings will be discussed. First, normality tests were conducted for all scales used in this study. For the vast majority of the variables, a normal distribution of scores was found. In fact, the only exception was the RCADS depression subscale, for which a skewness of 1.48 and kurtosis of 2.36 was documented. A transformation of the data on this measure only led to minimal changes in the results and therefore it was decided to report findings using the original scores. Second, the reliability coefficients of both questionnaires of self-conscious emotions were good. More precisely, Cronbach’s alphas were 0.79 for the shame proneness and 0.76 for the guilt proneness scales of the TOSCA-C, whereas alphas ranging between 0.74 and 0.80 were obtained for shame and guilt scales of child, parent and teacher versions of the BSGQ. The psychopathology questionnaires showed moderate good internal consistency, with alphas ranging between 0.55 and 0.94. Third, independent samples *t*-tests revealed a number of statistically significant gender differences. With regard to self-conscious emotions, girls scored higher on the shame proneness [child: *t*(155) = 5.12, *p* < 0.001, *d* = 0.83] and the guilt proneness subscales of the BSGQ-C [child: *t*(155) = 3.27, *p* < 0.001, *d* = 0.53; parent: *t*(151) = 2.46, *p* < 0.05, *d* = 0.42; teacher: *t*(125) = 2.08, *p* < 0.05, *d* = 0.39]. Furthermore, boys displayed higher levels of externalizing problems than girls on the YSR, CBCL, and TRF [child: *t*(157) = 2.65, *p* < 0.01, *d* = 0.43; parent: *t*(94.30) = 3.83, *p* < 0.001, *d* = 0.66; teacher: *t*(74.02) = 3.49, *p* < 0.001, *d* = 0.67]. Similar findings were found on the AQ: boys showed higher levels of ADHD as compared to girls [child: *t*(157) = 3.35, *p* < 0.001, *d* = 0.54; parent: *t*(90.39) = 3.22, *p* < 0.01, *d* = 0.56]. Boys displayed higher levels of aggression on the RPQ [*t*(154) = 3.43, *p* < 0.001, *d* = 0.55] as compared to girls. Finally, the effect of age was explored by means of correlation analyses. Shame proneness as measured by the BSGQ-C was significantly negatively associated with age (*r* = −0.21, *p* < 0.01), which implies that with increasing age the shame scores on this measure tended to decrease. A negative correlation was also found between age and parent-reported ADHD symptoms (*r* = −0.17, *p* < 0.05), suggesting that these symptoms were less notable in older children. Borderline personality features as measured by the BPFSC-11 were positively associated with age (*r* = 0.16, *p* < 0.05), indicating that with increasing age borderline personality features tended to become more prominent.

### Comparisons of Self-Conscious Emotions Between Clinical and Non-clinical Youths

A series of analyses of variance were conducted to evaluate whether there were statistically significant differences between the clinical and non-clinical children with regard to both explicit and implicit levels of shame and guilt proneness. In these analyses, age and gender were included as covariates (ANCOVAs) as there were some significant effects of these demographic variables on self-conscious emotions.

#### Explicit Shame and Guilt

The results of the comparisons of explicit self-conscious emotions between clinical and non-clinical children are shown in the two upper panels of Figure 1. As can be seen, on the TOSCA-C, no significant group differences were found [both *F*(1,155)’s < 1]. Note, however, that in general clinical and non-clinical children reported higher levels of guilt proneness than shame proneness on the TOSCA-C [paired *t*(29) = 9.70, *p* < 0.001, *d* = 1.86 in the clinical group, and paired *t*(128) = 21.97, *p* < 0.001, *d* = 1.88 in the non-clinical group]. The second explicit measure of self-conscious emotions, the BSGQ-C, was completed by children and parents of both groups. When looking at the child self-report data, it was found that participants in the non-clinical group reported higher levels of guilt proneness on the BSGQ-C than those in the clinical group [*F*(1,153) = 5.30, *p* < 0.05, *d* = 0.42]. The analyses of parent-report BSGQ-C data yielded significant effects for both shame and guilt proneness [*F*(1,149) = 5.33, *p* < 0.05, *d* = 0.53 and *F*(1,149) = 10.35, *p* < 0.01, *d* = 0.74, respectively]: parents of non-clinical children reported higher levels of both self-conscious emotions for their offspring than parents of the clinical group children.

#### Implicit Shame and Guilt

The data of 10 participants (five in the clinical and five in the non-clinical group) were excluded from the data analysis because of a too high error rate. Mean D600-scores of the shame- and guilt-related IATs of clinical and non-clinical children are displayed in the bottom panel of Figure 1. Contrary to our hypothesis, no between-group differences were found with regard to levels of implicit shame and guilt, with both *F*(1,149)’s < 1. Note that the D600 scores of both groups were negative, which implies that in general the children were quicker in pairing themselves with the positive attributes of pride than with attributes related to shame and guilt. Further, the results of additional paired samples *t*-tests showed that, in the non-clinical group, D600 scores for the guilt IAT were significantly smaller than those found for the shame IAT [*t*(123) = 3.00, *p* < 0.01, *d* = 0.31]. This suggests that non-clinical youths had relatively more difficulty to associate themselves with attributes of guilt than to relate themselves to attributes of shame. Within the clinical group, no significant difference in the D600 scores of both IAT versions was found [*t*(24 < 1].

### Correlations Among Implicit and Explicit Shame and Guilt Scores

Partial correlation coefficients (corrected for gender and age) among children’s explicit and implicit shame and guilt scores were calculated (Table 2). Moderate and statistically significant correlations were found between the shame and guilt proneness scales of the TOSCA-C (*r* = 0.51, *p* < 0.001) and the BSGQ-C (*r* = 0.44, *p* < 0.001), indicating that both self-conscious emotions are to some extent interrelated. Further, the shame and guilt proneness subscales of the TOSCA-C were significantly positively related with their counterpart subscales of the BSGQ-C (*r* = 0.52, *p* < 0.001 and *r* = 0.45, *p* < 0.001, respectively). Shame proneness as measured with the BSGQ-C was also significantly and positively associated with guilt proneness of the TOSCA-C, although this correlation was clearly more modest (*r* = 0.26, *p* < 0.001). Shame proneness of the BSGQ-C, completed by the children, showed a significant and positive association with parent-reported shame proneness (*r* = 0.33, *p* < 0.001), but no significant association with teacher-reported shame proneness. Child-reported guilt proneness of the BSGQ-C was not significantly correlated with parent- and teacher-reported BSGQ-C guilt proneness. Finally, implicit scores of shame and guilt (i.e., the IAT D600 scores) were not significantly correlated with the explicit measures of self-conscious emotions (i.e., TOSCA-C and BSGQ-C). Results did show that the D600 scores of shame IAT and guilt IAT were positively correlated with each other (*r* = 0.31, *p* < 0.001).

**TABLE 2:** Correlations (controlled for gender and age) between explicit and implicit measures of self-conscious emotions as completed by the children (*N* = 154).

	TOSCA-C shame	TOSCA-C guilt	BSGQ-C shame	BSGQ-C guilt	IAT shame
TOSCA-C shame					
TOSCA-C guilt	0.51*				
BSGQ-C shame	0.52*	0.26*			
BSGQ-C guilt	0.27*	0.45*	0.44*		
IAT shame	–0.06	–0.02	–0.09	0.05	
IAT guilt	–0.06	0.07	–0.02	–0.01	0.31*

BSGQ-C, Brief Shame and Guilt Questionnaire for Children; IAT, Implicit Association Test; TOSCA-C, Test of Self-Conscious Affect for Children. *p < 0.001.

### Shame, Guilt, and Psychopathological Symptoms

Because differences in self-conscious emotions between the clinical and non-clinical groups were not as substantial as anticipated and because the sample size of the clinical group was rather small, it was decided to perform our planned correlational analysis in the total sample of children and adolescents^1^. Partial correlations (controlled for gender and age) between the explicit measures of self-conscious emotions (i.e., TOSCA-C and BSGQ-C) and the questionnaires assessing various types of psychopathological symptoms are shown in Table 3. Three general conclusions can be drawn from these results. First, many of the observed significant correlations were positive, indicating that higher levels of shame and guilt proneness were associated with higher levels of symptoms. However, there were also some significant negative correlations implying that proneness to these self-conscious emotions was accompanied by lower symptom levels, and this was especially the case in correlations involving guilt proneness and/or externalizing problems. Second, shame proneness showed more robust correlations with symptoms of psychopathology than guilt proneness. Third, within-informant correlations were more robust as compared to correlations computed across informants. Below we will discuss the results of these partial correlations analyses in somewhat more detail.

**TABLE 3:** Correlations (controlled for gender and age) between the child- and parent-report scales measuring the self-conscious emotions of shame and guilt and various indices of psychopathology as completed by the children (upper panel), parents (middle panel), and teachers (lower panel).

	Shame			Guilt		
Child	TOSCA-C (child)	BSGQ-C (child)	BSGQ-C (parent)	TOSCA-C (child)	BSGQ-C (child)	BSGQ-C (parent)
*N*	154	154	149	154	154	149
AQ ADHD	0.16*	0.03	–0.05	0.01	–0.15	−0.18*
BPFS-C borderline	0.46***	0.24**	0.11	0.16	0.05	0.02
RCADS-25 anxiety	0.51***	0.43***	0.23**	0.37***	0.23**	0.13
RCADS-25 depression	0.29***	0.19*	0.13	0.13	0.01	0.07
RPQ aggression	0.04	–0.04	–0.11	–0.06	−0.23***	−0.16*
YSR internalizing	0.40***	0.32***	0.20*	0.12	0.07	0.14
YSR externalizing	0.16*	0.01	–0.12	–0.03	−0.19*	−0.22**
**Parent**						
*N*	151	149	151	151	149	151
AQ ADHD	0.05	–0.06	–0.01	0.04	–0.15	–0.14
RCADS-25 anxiety	0.28***	0.16	0.34***	0.14	0.12	0.06
RCADS-25 depression	0.15	0.10	0.08	0.02	0.06	–0.01
CBCL internalizing	0.11	0.11	0.10	0.06	–0.07	–0.04
CBCL externalizing	0.02	0.00	–0.06	–0.01	–0.13	−0.25**
**Teacher**						
*N*	125	123	122	125	123	122
TRF internalizing	0.22*	0.13	0.28**	0.17	0.12	0.23*
TRF externalizing	–0.04	–0.05	0.02	–0.10	–0.11	–0.05

*AQ, ADHD-Questionnaire; BPFS-C, Borderline Personality Features Scale for Children; CBCL, Child Behavior Checklist; RCADS-25, Revised Child Anxiety and Depression Scale; RPQ, Reactive-Proactive aggression Questionnaire; TOSCA-C, Test of Self-Conscious Affect for Children; TRF, Teacher Report Form; YSR, Youth Self-Report.*

*p < 0.05, **p < 0.01, ***p < 0.001.

#### Psychopathological Correlates of Child-Reported Self-Conscious Emotions (Test of Self-Conscious Affect for Adolescents and Brief Shame and Guilt Questionnaire for Children)

Shame proneness as measured by means of the TOSCA-C was significantly and positively associated with ADHD symptoms, borderline personality features, anxiety and depression symptoms, and internalizing and externalizing problems as reported by the children (all *r*s between 0.16, *p* < 0.05 and 0.51, *p* < 0.001). Guilt proneness was less convincingly associated with psychopathology symptoms: only one significant positive correlation was found with the anxiety scale of the RCADS-25 (*r* = 0.37, *p* < 0.001). Correlations between TOSCA-C scores and psychopathology symptoms as reported by parents were also less substantial, with the only statistically significant correlation being that between shame proneness and RCADS-25 anxiety (*r* = 0.28, *p* < 0.001). Shame proneness was positively linked to internalizing problems as reported by teachers (*r* = 0.22, *p* < 0.05).

Partial correlations computed between the BSGQ-C as completed by the children and psychopathological symptoms yielded a comparable pattern of results as was obtained with the TOSCA-C. That is, BSGQ-C shame proneness was significantly and positively associated with borderline personality features, anxiety and depression symptoms, and internalizing problems as reported by the children (all *r*’s between 0.19, *p* < 0.05 and 0.43, *p* < 0.001). Guilt proneness as measured by the child version of the BSGQ-C showed a significant and positive correlation with RCADS-25 anxiety symptoms (*r* = 0.23, *p* < 0.01), whereas significant negative correlations were found with RPQ aggression (*r* = −0.23, *p* < 0.01) and YSR externalizing (*r* = −0.19, *p* < 0.05). Partial correlations between child-reported BSGQ-C shame and guilt scores and measures of psychopathology as completed by parents and teachers were all non-significant.

#### Psychopathological Correlates of Parent-Reported Self-Conscious Emotions (Brief Shame and Guilt Questionnaire for Children)

Parent-report BSGQ-C shame proneness was significantly and positively associated with RCADS-25 anxiety symptoms (*r* = 0.23, *p* < 0.01) and internalizing problems (*r* = 0.20, *p* < 0.05) as reported by the children. Guilt proneness as measured by the parent version of the BSGQ-C was significantly and negatively associated with ADHD symptoms (*r* = −0.18, *p* < 0.05), aggression (*r* = −0.16, *p* < 0.05), and externalizing problems (*r* = −0.22, *p* < 0.01) as reported by children. Partial correlations between parent-reported BSGQ-C scores and measures of psychopathology as completed by the parents were mostly non-significant. Exceptions were shame proneness, which was positively linked to anxiety problems (*r* = 0.34, *p* < 0.001), and guilt proneness, which was negatively linked to externalizing problems (*r* = −0.25, *p* < 0.001). Correlations between parent-report BSGQ-C scores and psychopathology symptoms as reported by teachers revealed a significant and positive association between both shame proneness (*r* = 0.28, *p* < 0.01) and guilt proneness (*r* = 0.23, *p* < 0.05) and internalizing problems.

#### Psychopathological Correlates of Implicit Self-Conscious Emotions

Implicit self-conscious emotions were not significantly associated with psychopathological problems as reported by children and their parents. Thus, the shame and guilt scores of the IAT were not significantly correlated with more general (CBCL, YSR) or specific indices of psychopathology, and these also included child-reported borderline features as measured with the BPFS-C (shame: *r* = −0.11, *p* = 0.18; guilt: *r* = −0.03, *p* = 0.69).

## Discussion

In general, research in children and adolescents has indicated that higher levels of shame proneness and (to a lesser extent) guilt proneness are associated with higher levels of all kinds of psychopathological symptoms, while lower levels of guilt proneness are accompanied by higher levels of externalizing problems (Muris and Meesters, 2014). However, previous studies have mainly relied on non-clinical populations and predominantly used explicit self-report measurements of self-conscious emotions. The present study adopted a multi-informant, multi-method approach to study psychopathological correlates of shame and guilt proneness in clinical and non-clinical youths aged 8–17 years. Both self-conscious emotions were measured explicitly via two self-reports (TOSCA-C and BSGQ-C) and a parent-report (BSGQ-C), and implicitly by means of an IAT, while a wide range of psychopathological symptoms were assessed with questionnaires completed by children, parents, and teachers.

Based on reviews of the literature (e.g., Tangney et al., 1992b; Muris and Meesters, 2014), it was hypothesized that clinically referred youths would display more dysregulated levels of shame and guilt, both on explicit and implicit measures, as compared to non-clinical youths. A first conclusion that can be drawn from the present study is that this hypothesis was only partly supported by the data. That is, the young participants in the clinical group did not show *higher* levels of shame and guilt on any of the included explicit and implicit measures of self-conscious emotions (i.e., TOSCA-C, BSGQ-C child- and parent-report, and IAT) than the participants in the non-clinical group. The main reason for this unexpected finding seems to be that the clinically referred youth, in comparison to their non-clinical counterparts, most clearly showed elevated symptom levels of an externalizing nature: that is, on both self- and parent-report measures of disruptive behavior problems the clinical group displayed significantly higher scores than the non-clinical group. With regard to internalizing symptoms the differences between both groups were less prominent: there were some significant differences with a small effect size on the parent-report and no statistically significant differences on any of the child reports. Given that the presence of high levels of anxiety and depression have been found to be a driving force behind high levels of self-conscious emotion (De Rubeis and Hollenstein, 2009; Muris et al., 2015), it is not that surprising that we did not obtain heightened levels of shame and guilt in this specific clinical group of children and adolescents.

Meanwhile, it was noted that clinically referred children and adolescents did exhibit *lower* levels of guilt (child- and parent-report) and shame (parent-report) on the BSGQ-C than youth in the non-clinical control group. This suggests that the youngsters in our clinical population were more typified by deficits in experiencing self-conscious emotions rather than excessive levels of shame and guilt. Although we need to be cautious with interpreting this result as it was not found on the other measures of self-conscious emotions (TOSCA-C, IAT), it seems most plausible to ascribe this finding to the fact that (unintentionally) our sample predominantly consisted of youngsters with a diagnosis of ADHD (i.e., 70%). Previous research has indicated that children with this type of neurodevelopmental disorder show deficits in the processing of emotions: they appear to perform less well on facial emotion recognition tasks and have more difficulties to discern emotions on the basis of contextual cues (Da Fonseca et al., 2009). Both of these processes seem to be highly relevant in the formation of self-conscious emotions, and so it makes sense that in particular youths with ADHD are less susceptible to experience them (Scheel et al., 2014; Muris et al., 2016; Castagna et al., 2021), resulting in deficits of interpersonal sensitivity and social difficulties (Harpin, 2005).

To use the full variance in self-conscious emotions and psychopathology scores, we also conducted correlational analyses to explore the relations between guilt and shame and symptoms of various disorders as reported by the three informants (i.e., child, parent, and teacher) within the total sample of children and adolescents. In general, the pattern of results was quite similar across informants, but the strongest correlations were noted in the data that were provided by youths themselves. This seems logical because shame and guilt refer to emotional responses that have a secretive or hidden nature and that hence often remain unnoticed for other people such as parents and teachers (Verhulst and Van der Ende, 1992; De Los Reyes and Kazdin, 2005). To simplify the discussion of these results, we will first and foremost focus on the child-report data here. Findings revealed that higher levels of child-reported shame, either measured by the TOSCA-C or the BSGQ-C, were associated with higher levels of a broad range of internalizing psychopathology, with most robust correlations being noted for anxiety symptoms, followed by borderline features and depression. This confirms previous studies showing that heightened levels of shame are a prominent feature of anxiety (Muris et al., 2018; Irwin et al., 2019; Broekhof et al., 2020; Sullivan et al., 2020), personality (Hawes et al., 2013; Wall et al., 2021) and depressive (Feiring et al., 2002; Tilghman-Osborne et al., 2008; Bennett et al., 2010) psychopathology: all these psychopathologies are characterized by strong (social) avoidance and it seems plausible that high levels of shame fuel such evading and oftentimes submissive responses.

Child-reported guilt was also positively associated with anxiety symptoms, but not with other types of internalizing psychopathology, although these correlations were of a smaller magnitude than those found for shame, which is also in line with other studies showing that shame is the more toxic self-conscious emotion and that guilt only becomes maladaptive when experienced in a ruminative manner (e.g., Taihara and Malik, 2016). Guilt as measured with the child-report BSGQ-C, but not when assessed with the TOSCA-C, was also to some extent negatively correlated with externalizing psychopathology (i.e., aggression and YSR externalizing). This finding is in line with other research showing that lower levels of guilt can be noted in youth displaying disruptive behavior problems (e.g., Stuewig and Tangney, 2007; Stuewig et al., 2010), and seems to support the notion that guilt is the more ‘positive’ self-conscious emotion that drives empathy, affiliation, and prosocial behavior and that those who lack this emotion are prone to display antagonistic, aggressive, and antisocial behavior (Stuewig and Tangney, 2007).

As already alluded to above, parent-reported self-conscious emotions were less clearly related to youths’ psychopathology scores. However, the few statistically significant correlations that were documented were generally in keeping with what was found with the child-report measures of shame and guilt. More precisely, parent-reported shame was positively correlated with child- and parent-reported anxiety symptoms and with child- and teacher-reported internalizing symptoms. Parent-reported guilt, on the other hand, was mainly negatively associated with child-reported aggression, externalizing symptoms, and ADHD as well as with parent-reported externalizing symptoms. Together, these findings confirm the relevance of shame in the study of anxiety and emotional psychopathology, while (lack of) guilt seems to be more pertinent in the study of externalizing and disruptive behavior problems.

No statistically significant correlations were found between implicit shame and guilt as measured with the IAT and the various measures of psychopathology. So far, few investigations examined this topic in youth samples, but on the basis of the Hawes et al. (2013) study, we expected to find a significant association between implicit shame and borderline personality features, but apparently this was not the case. On the one hand, it may well be that this was due to the fact that borderline symptom levels were rather low in this specific sample, which consisted of non-clinical youth as well as clinical youth who mainly had a diagnosis of ADHD. On the other hand, there may have been methodological issues with the IAT version that was developed for the purpose of the present study. In general, the IAT produced negative D600 values, which indicates that there was a stronger association with the attribute ‘pride’ than with the self-conscious emotions of ‘shame’ and ‘guilt’. Further, although the IAT intended to make a valid distinction between implicit shame and guilt, there are doubts on whether this attempt was really successful. In specific, it was quite surprising to note that the non-clinical youths associated themselves more easily with attributes of shame than with attributes of guilt. One explanation might be found in the words used in the contrasting attribute category of pride in both IAT tasks do not seem to tap the same concept. To be more specific, words of pride in the guilt IAT were more related to acting in a well-behaved manner, whereas words of pride in the shame IAT were indicative of excellent personal characteristics. This might have caused a different contrast and thus an unequal comparison of both self-conscious emotions.

Although the IAT that was used in the current study was based on the measurement procedure described by Hawes et al. (2013), a critical note should be made regarding the validity of this test as a measure of (implicit) self-conscious emotions. Shame and guilt are complex in nature: these emotions occur when a person makes a self-related cognitive attribution following a negative identity-relevant event, which typically reflects rejection or the threat of losing face (Muris and Meesters, 2014). Single word stimuli like ‘bad, stupid, loser, worthless’ (shame) or ‘fault, wrong naughty, disobedient’ (guilt) clearly lack such complexity and so one may wonder whether they truly reflect self-conscious emotions or primarily represent more simple constructs (such as ‘self-criticism’ and ‘defiance’).

With regard to various instruments that were used to assess the self-conscious emotions of shame and guilt, the TOSCA-C, BSGQ-C, and the IAT, a number of remarks can be made. To begin with, when looking at the correlations between scores on the two explicit measures, the TOSCA-C and the BSGQ-C, it can be concluded that their correspondence was at best modest. More specially, shame scores of both self-reports correlated 0.52, while guilt scores of both measures correlated 0.45, which means that percentages of shared variance were only 27 and 20%, respectively. Although both instruments intend to measure dispositional levels of shame and guilt, there are also clear differences between them. The TOSCA-C is a vignette-based measure which provides clear response options that reflect young people’s possible cognitive interpretations in hypothetical situations that typically elicit self-conscious emotion. Based on Lewis’ (2000) pioneering work, the response option for guilt describes the child realizing doing something wrong that may call for a reparative action, whereas the response option for shame depicts the child who after a transgression blames himself of being a worthless person. The BSGQ-C also makes use of scenarios but here children and adolescents are simply asked to rate their levels of shame and guilt in these situations. This procedure assumes that young people understand these self-conscious emotions and can make a proper distinction between shame and guilt. The developmental psychology literature suggests that children from 7 years onwards have sufficient knowledge about these emotions, but it has also been noted that the depth of this understanding still varies considerably as a function of age (Ferguson et al., 1991; Berti et al., 2000). Furthermore, the cross-informant correlations that were obtained for the BSGQ-C were all small and negligible. Only one correlation attained statistical significance: child-reported shame on this measure was positively related to parent-reported shame. This result provides a further illustration of the fact that the self-conscious emotions of shame and guilt are internal and private experiences that are mostly not readily observed by other people in youths’ direct environment such as parents and teachers. Finally, implicit shame and guilt as assessed by the IAT were not significantly correlated with explicit measurements of these self-conscious emotions. In their comprehensive review on implicit measures of association, Roefs et al. (2011) noted this finding is not unusual. It illustrates that implicit and explicit measures tap different aspects of an underlying construct. In particular, when the assessed construct refers to a cognitive evaluation of a continuous personality characteristic, the implicit-explicit correspondence can be expected to be rather low. Still, the absence of a correlation between the IAT and the shame/guilt scales in our study raise questions about the validity of the data.

The present study also yielded a number of additional findings that deserve some brief comments. First, the gender effects that were found were largely in line with what has been documented in the literature. That is, girls experienced higher levels of shame and guilt than boys, which is in correspondence with previous research demonstrating that females are more susceptible to these self-conscious emotions than males (Else-Quest et al., 2012). Second, a significant age effect was noted indicating that shame tended to decrease with increasing age. This finding is not in line previous results implying that there is an age-related progression in the experience of self-conscious emotions (Leverato and Donati, 1999; Berti et al., 2000; Olthof et al., 2000). Note, however, that this age effect was only found with the BSGQ-C and perhaps can best be interpreted in the light of the earlier mentioned abstract nature of the scale. At a younger age, children may have more difficulty to understand the exact nature of shame and hence may provide somewhat inflated ratings of this self-conscious emotion. In older children, the knowledge of these emotions has increased and this is expressed in more realistic (lower) ratings of shame. Third, there were also gender and age effects for a number of psychopathological problems. Boys reported more ADHD and externalizing problems than girls, which is in line with previous studies (Costello et al., 2003). With regard to the age differences, it was found that as children get older, fewer ADHD problems but more borderline personality problems were reported. This result is also consistent with previous studies (e.g., Guilé et al., 2018; Holland and Sayal, 2018).

### Limitations

It should be acknowledged that the present study suffers from various limitations. The most important shortcoming was that the clinical group was quite small and mainly consisted of children and adolescents with a diagnosis of ADHD. The clinical group unexpectedly contained relatively few young participants with anxiety disorders, depression, and borderline personality problems, which are types of psychopathology for which we considered shame and guilt as particularly relevant. A further shortcoming was that we did not include scales for measuring eating disorders and autism spectrum disorder, which are two other types of psychopathology in which dysregulated self-conscious emotions have been shown to play an important role (Heerey et al., 2003; Cavalera et al., 2016; Muris et al., 2016). Another demerit pertains to the cross-sectional design of the study: in both clinical and non-clinical children, data on self-conscious emotions and psychopathology were collected at one point in time, which implies that it is not possible to interpret the results in terms of cause-effect relations. A final drawback concerned the quite extensive survey of self-report scales that had to be completed by the children and adolescents. In particular, for youth in the clinical group who already were subjected to several diagnostic procedures and younger children in the non-clinical group. This may have had a negative impact on the participation and drop-out rates.

Future research should include a larger group of both clinical children and adolescents with a more balanced mix of internalizing and externalizing disorders as well as make an attempt to include multiple measurements in order to follow youth for a longer period of time, allowing to draw causal conclusions about the role of self-conscious emotions in the development of psychopathology. The present study has indicated that the link between shame and guilt and psychopathology is complex, and at least critically depends on the type of disorder under study. Moreover, there is increasing evidence that there are all kinds of context-related factors that determine the maladaptive nature of these self-conscious emotions (e.g., Taihara and Malik, 2016) thereby framing its contribution to psychopathology in children and adolescents (Muris and Meesters, 2014). With more of such insights, treatments for various forms of psychopathology can be developed that also aim to correct the dysregulation of guilt and shame, which may have better efficacy than interventions that solely focus on the abolishment of extreme basic emotions.

## Data Availability Statement

The raw data supporting the conclusions of this article will be made available by the authors, without undue reservation, to any qualified researcher.

## Ethics Statement

The studies involving human participants were reviewed and approved by Ethical Research Committee of Psychology and Neuroscience, Maastricht University. Written informed consent to participate in this study was provided by the participants’ legal guardian/next of kin.

## Author Contributions

EH, PM, and CM designed the study. EH designed the computer task and survey, collected and processed the data, conducted the statistical analyses, and wrote the article. PM and CM supervised the data collection and processing and assisted in writing the article. KH assisted with the analyses of the computer data. All authors contributed to the article and approved the submitted version.

## Conflict of Interest

The authors declare that the research was conducted in the absence of any commercial or financial relationships that could be construed as a potential conflict of interest.

## Publisher’s Note

All claims expressed in this article are solely those of the authors and do not necessarily represent those of their affiliated organizations, or those of the publisher, the editors and the reviewers. Any product that may be evaluated in this article, or claim that may be made by its manufacturer, is not guaranteed or endorsed by the publisher.

## Footnotes

1 We also conducted these correlational analyses for the clinical and non-clinical group separately. Results showed more significant correlations between self-conscious emotions and indices of psychopathology in the non-clinical group than the clinical group, which was probably due to the larger sample size of the former group. The pattern of correlations was quite similar in both groups.
