# Supplementary material for: Childhood Disorder: Dysregulated Self-Conscious Emotions? Psychopathological Correlates of Implicit and Explicit Shame and Guilt in Clinical and Non-clinical Children and Adolescents
Source: Front Psychol. 2022 Mar 9;13:822725. doi: 10.3389/fpsyg.2022.822725 (PMC8959856; doi:10.3389/fpsyg.2022.822725)
Supplement: Supplementary file 1 [file Table_1.docx]

# **Supplementary material**


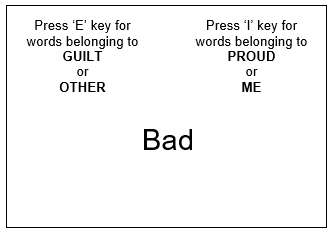

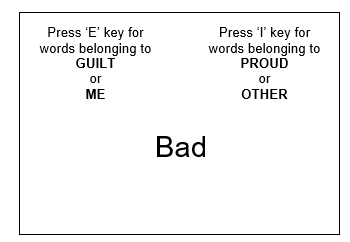
**Figure 1**
Administration of the implicit association test for guilt-prone self-concept in block 3 (left) and block 5 (right). In the shame-prone self-concept words of guilt were replaced with words of shame.

Note. Example of the Guilt Implicit Association Test
